# Atomic and Electronic Structure in MgO–SiO_2_

**DOI:** 10.1021/acs.jpca.3c05561

**Published:** 2024-01-18

**Authors:** Yuta Shuseki, Shinji Kohara, Tomoaki Kaneko, Keitaro Sodeyama, Yohei Onodera, Chihiro Koyama, Atsunobu Masuno, Shunta Sasaki, Shohei Hatano, Motoki Shiga, Ippei Obayashi, Yasuaki Hiraoka, Junpei T. Okada, Akitoshi Mizuno, Yuki Watanabe, Yui Nakata, Koji Ohara, Motohiko Murakami, Matthew G. Tucker, Marshall T. McDonnell, Hirohisa Oda, Takehiko Ishikawa

**Affiliations:** † Graduate School of Engineering, Kyoto University, Kyoto 615-8520, Japan; ‡ Center for Basic Research on Materials, National Institute for Materials Science (NIMS), Tsukuba, Ibaraki 305-0047, Japan; § Department of Computational Science and Technology, Research Organization for Information Science and Technology (RIST), Tokyo 105-0013, Japan; ∥ Human Spaceflight Technology Directorate, Japan Aerospace Exploration Agency (JAXA), Tsukuba, Ibaraki 305-8505, Japan; ⊥ Graduate School of Science and Technology, Hirosaki University, Hirosaki, Aomori 036-8561, Japan; # Unprecedented-Scale Data Analytics Center, Tohoku University, Sendai, Miyagi 980-8578, Japan; ∇ Graduate School of Information Science, Tohoku University, Sendai, Miyagi 980-8579, Japan; ○ RIKEN Center for Advanced Intelligence Project, Tokyo 103-0027, Japan; ◆ Center for Artificial Intelligence and Mathematical Data Science, Okayama University, Okayama 700-8530, Japan; ¶ Institute for the Advanced Study of Human Biology (WPI-ASHBi), Kyoto University, Kyoto 606-8303, Japan; & Institute for Materials Research, Tohoku University, Sendai, Miyagi 980-8577, Japan; ● National Institute of Technology, Hakodate College, Hakodate, Hokkaido 042-8510, Japan; ◊ Advanced Engineering Services Co., Ltd., Tsukuba, Ibaraki 305-0032, Japan; ▲ Faculty of Materials for Energy, Shimane University, Matsue, Shimane 690-8504, Japan; □ Department of Earth Sciences, ETH Zürich, Zürich 8092, Switzerland; ▼ Neutron Scattering Division, Oak Ridge National Laboratory, Oak Ridge, Tennessee 37831, United States; ¢ Computer Science and Mathematics Division, Oak Ridge National Laboratory, Oak Ridge,Tennessee 37830, United States; + Institute of Space and Astronautical Science, Japan Aerospace Exploration Agency (JAXA), Tsukuba, Ibaraki 305-8505, Japan

## Abstract

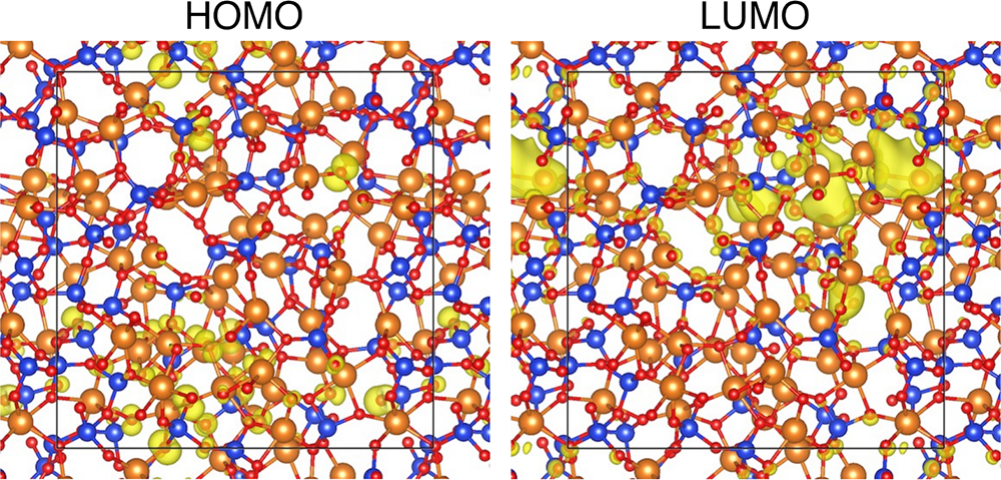

Understanding disordered structure is difficult due to insufficient information in experimental data. Here, we overcome this issue by using a combination of diffraction and simulation to investigate oxygen packing and network topology in glassy (*g*-) and liquid (*l*-) MgO–SiO_2_ based on a comparison with the crystalline topology. We find that packing of oxygen atoms in Mg_2_SiO_4_ is larger than that in MgSiO_3_, and that of the glasses is larger than that of the liquids. Moreover, topological analysis suggests that topological similarity between crystalline (*c*)- and *g*-(*l*-) Mg_2_SiO_4_ is the signature of low glass-forming ability (GFA), and high GFA *g*-(*l-*) MgSiO_3_ shows a unique glass topology, which is different from *c-*MgSiO_3_. We also find that the lowest unoccupied molecular orbital (LUMO) is a free electron-like state at a void site of magnesium atom arising from decreased oxygen coordination, which is far away from crystalline oxides in which LUMO is occupied by oxygen’s 3*s* orbital state in *g*- and *l*-MgO–SiO_2_, suggesting that electronic structure does not play an important role to determine GFA. We finally concluded the GFA of MgO–SiO_2_ binary is dominated by the atomic structure in terms of network topology.

## Introduction

The MgO–SiO_2_ system is very important in both glass science and geoscience^1^ since glassy (*g*)-MgO–SiO_2_ is a typical binary silicate glass system and crystalline (*c*)-MgSiO_3_ (enstatite) and *c*-Mg_2_SiO_4_ (forsterite) are Mg-end members of main components of the Earth’s mantle. Liquid (*l*)-Mg_2_SiO_4_ can be classified as a fragile liquid, while *l*-MgSiO_3_ is a stronger liquid according to Angell.^2^ Particularly, viscosity under high pressure and high temperature is an important thermophysical property to understand magma ocean solidification.^3^ The structures of *g*-MgSiO_3_ (high glass-forming ability (GFA)) and *g*-Mg_2_SiO_4_ (low GFA) have been widely studied because the use of the levitation technique^4^ made it possible to synthesize a bulk *g*-Mg_2_SiO_4_.^5^ Numerous studies using X-ray^6−9^ and neutron^7−9^ diffraction, NMR,^5,10−13^ Raman spectroscopy,^14^ and reverse Monte Carlo (RMC)^15^–density functional (DF) theory simulation have been reported.^9^ The structure of liquid (*l*)-MgSiO_3_ has been studied by X-ray diffraction^16,17^ and DF–molecular dynamics (MD) simulation.^9^ In the case of *l*-Mg_2_SiO_4_, available data are very limited due to a high melting point (1850 °C); only synchrotron X-ray diffraction data^17^ are available, while DF–MD simulation data are reported.^9^ The previous diffraction studies report that SiO_4_ tetrahedra are stable, and the Mg–O coordination number (CN) is around 5 in *l*- and *g*-MgO–SiO_2_, although there are some discrepancies in Mg–O CN’s in the previous reports. NMR spectroscopy confirmed that the *Q*^2^ species (SiO_4_ chain) are dominant in *g*- and *l*-MgSiO_3_, while Si_2_O_7_^6–^ dimers and isolated SiO_4_^4–^ are dominant in *g*- and *l*-Mg_2_SiO_4_.

In this article, we performed high-energy X-ray diffraction and neutron diffraction measurements on *l*-MgSiO_3_ and *l*-Mg_2_SiO_4_ to obtain more detailed structural information about the liquids. To obtain atomic configurations with detailed electronic structures of *g*- and *l*-MgO–SiO_2_, advanced DF–MD simulations for *g*- and *l*-MgO–SiO_2_ were conducted to understand the electronic structure in MgO–SiO_2_. We measured the density of *l*-Mg_2_SiO_4_ by using an electrostatic levitation furnace (ELF) under microgravity at the International Space Station (ISS). Moreover, we performed several topological analyses (ring, polyhedral connection analysis, and persistent homology) on crystalline (*c*-), *g*-, and *l*-MgO–SiO_2_ to extract topological similarity among the crystal, glass, and liquid to understand the relationship between the topology and GFA.

## Experimental and Simulation Procedures

Stoichiometric mixtures of MgO and SiO_2_ were annealed in air in 12 h to obtain polycrystalline MgSiO_3_ and Mg_2_SiO_4_ for levitation experiments. Spherical samples with a diameter of 2–3 mm were prepared by melting the *c*-MgSiO_3_ and *c*-Mg_2_SiO_4_ using a CO_2_ laser heating on an aerodynamic levitator. Samples of *g*-MgSiO_3_ were obtained during cooling, while 2–3 mm Mg_2_SiO_4_ was too large to be vitrified because of its low GFA.

The density of *l*-MgO–SiO_2_ was measured with an ELF at the ISS. The following obstacles exist when measuring oxide materials using an ELF on the ground: (1) A large voltage is required to overcome the gravity, but this voltage is discharged. (2) A vacuum is required to avoid discharge, but in that case, the oxides will volatilize. On the other hand, the above two problems can be avoided in space; this is why the measurements must be performed on the ISS. A sample was 2 mm in diameter. It was charged by friction or contact with other materials in ISS–ELF^18^ and then levitated to the center between six electrodes that applied Coulomb force. The sample position was stabilized by tuning voltages between electrodes at 1000 Hz and monitoring the image of the sample backlit with a He–Ne laser. The levitated sample was heated and melted by four 40 W semiconductor lasers (980 nm) under 2 atm of dry air. The temperature of the sample was measured by a pyrometer (1.45–1.8 μm). It was calibrated using an emissivity calculated from the plateau temperature at recalescence and the reference value of the melting point (MgSiO_3_: 1650 °C and Mg_2_SiO_4_: 1850 °C^19^). After melting, the nonspherical sample became spherical upon cooling after shutting off the lasers. During cooling, the sample image was observed by ultraviolet back light and a CCD camera. The pixel size was calibrated against an image of 2.0 mm stainless steel spheres, which was recorded under the same conditions as the sample. The sample volume was calculated from its diameter obtained from the image. The density was calculated from the volume and weight measured by UMX2 (Metler TOLEDO) after the sample was returned to the earth.

The X-ray diffraction measurements of *l*-MgSiO_3_ and *l*-Mg_2_SiO_4_ were performed at the BL04B2 beamline^20^ of SPring-8 using an aerodynamic levitator.^21^ The energy of the incident X-rays was 61.4 keV. The 2 mm sample was levitated in dry air and heated by a 200 W CO_2_ laser. The temperature of the sample specimen was monitored by a two-color pyrometer (0.9 and 1.05 μm). The instrument background was successfully reduced by shielding the detectors and by optimizing a beam stop. The measured X-ray diffraction data were corrected for polarization, absorption, and background, and the contribution of Compton scattering was subtracted using standard analysis procedures.^22^

The neutron diffraction measurements were conducted on the Nanoscale-Ordered Materials Diffractometer (NOMAD) diffractometer^23^ at SNS of Oak Ridge National Laboratory using an aerodynamic levitator. The 3 mm sample was levitated in dry argon and heated by a 400 W CO_2_ laser. The temperature of the sample specimen was monitored by a two-color pyrometer. The measured scattering intensities for the samples were corrected for instrument background, absorption of the samples, and multiple and incoherent scattering and then normalized by the incident beam profile.

The fully corrected data sets were normalized to give the Faber–Ziman^24^ total structure factor *S*(*Q*), and the total correlation function *T*(*r*) was obtained by a Fourier transform of *S*(*Q*).

The initial configurations for *l*-MgO–SiO_2_ were generated by RMC modeling started with a random configuration using both X-ray and neutron diffraction data. The RMC++^25^ code was used. The number of particles in the unit cells was 510 and 511 for MgSiO_3_ and Mg_2_SiO_4_, respectively. DF–MD calculations were performed using the CP2K code,^26^ which is a software package for DF–MD calculations using the hybrid Gaussian (MOLOPT-DZVP-SR) and plane wave basis set. The generalized gradient approximation (GGA) of Perdew, Burke, and Ernzerhof^27^ was adopted for the exchange–correlation energy functional. The norm-conserving pseudopotentials of Goedecker, Teter, and Hutter^28^ were adopted. The cutoff energy of the plane wave was set to 400 Ry. *NVT* simulations were carried out using the Nose–Hoover chain method with three thermostats. We performed MD simulations for 20 ps with the time step of 1 fs at 293 K for glass and at 2073 K for liquid.

For the electronic structure calculations, we used the structures of DF–MD at 10 ps for glasses and 10 and 20 ps for liquids. We employed the PHASE/0 code,^29^ which is DF calculations using a plain wave basis set. The norm-conserving pseudo potentials^30^ were used for Mg and Si atoms, while ultrasoft pseudo potential^31^ was used for O atoms. The PBE0 hybrid functional^32^ with fraction α = 0.3 was used for much more reliable estimation of band gap, where Γ is the fraction of the exact exchange term in the functional. The *k*-sampling was 2 × 2 × 2 for the Γ point centered mesh with tetrahedron method. The cutoff energies of plane wave basis set and charge density were 25 and 225 Ry, respectively. For the evaluation of the exact exchange term, only the gamma point was sampled, and the real-space method was used for the deficit charge term.

**Figure 1:**
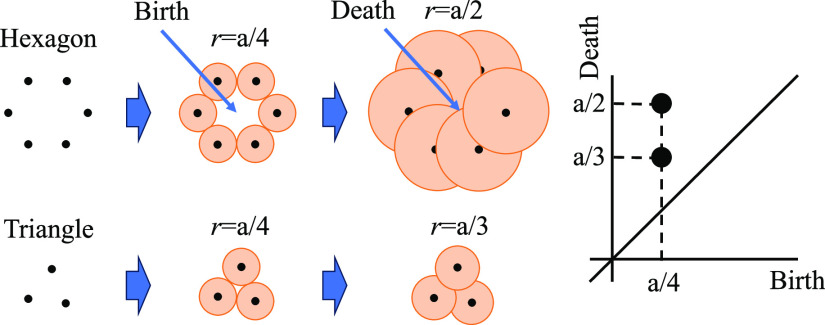
Methodology of the persistent diagram.

King ring size distributions were calculated by using R. I. N. G. S. code.^33^ The homology of atomic configurations for *c*-, *g*, and *l*-MgO–SiO_2_ was investigated using the PD_1_, which is comprised of two-dimensional histograms showing a persistent homology. Figure 1 shows the methodology of PD_1_.^34^*D*_1_ of a set of atoms given by the following thickening process of spheres: (i) place a sphere with a radius *r* at the center of each atom, (ii) increase the radii of the spheres from 0 to a sufficiently large value, and (iii) encode the pair of birth and death radii (*b*_*i*_, *d*_*i*_) for each ring *c*_*i*_ consisting of a set of spheres. The PD_1_ is then constructed by the two-dimensional histogram on the birth and death plane obtained by the pairs for independent *c*_*i*_, (*i* = 1,···, *K*). Here, the birth (death) radius is detected as the radius of spheres at which ring *c*_*i*_ first appears (disappears). The birth radius has information about the distances between atoms of the ring *c*_*i*_, and the death radius exhibits information about the size of the ring. The PD_1_ provides statistical information on the shapes of all rings and thereby provides insight into intermediate-range ordering in a disordered structure. The rings detected by this process are recorded for the computation of the PD_1_s; hence, their geometric shapes can be identified for further analyses. The PD_1_s were calculated using the HomCloud package.^35^

## Results and Discussion

### Density Data

**Figure 2:**
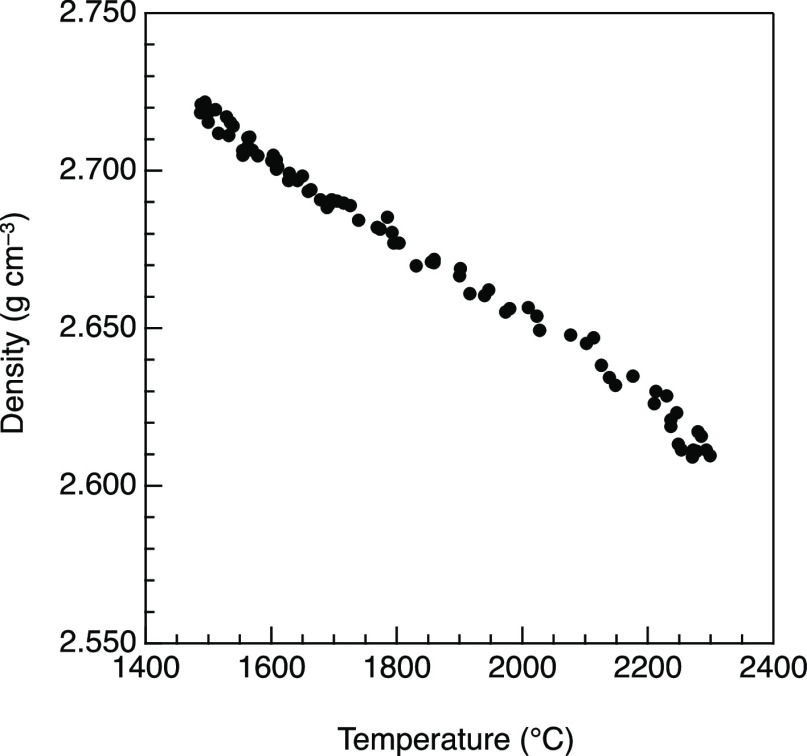
Measured density of *l*-Mg_2_SiO_4_ as a function of temperature. Error bar was estimated to be 3%.

Table 1 summarizes the published density data for MgO–SiO_2_. Note that density data for *l*-Mg_2_SiO_4_ is estimated in the supplemental data of ref (9). Density of *l*-Mg_2_SiO_4_ as a function of the temperature measured at the ISS using ELF is depicted in Figure 2. The density showed a linear temperature dependence, which can be fitted to
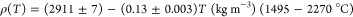
1with 99% confidence interval. The density is 2.678 g cm^–3^ at 1800 °C, very close to the estimated value of 2.677 g cm^–3^ given in Table 1. Experimental density data of *l*-*x*MgO–(100 – *x*)SiO_2_ (*x* = 30, 40, 50, 60, 66.7, 70) were also obtained (Figure S1). Both the densities for *g*- and *l*-Mg_2_SiO_4_ are higher than those of *g*- and *l*-MgSiO_3_, respectively. However, the density for *c*-Mg_2_SiO_4_ is comparable to that of *c*-MgSiO_3_ despite the increase in MgO content. The densities of the liquids are lower than those for the glasses, which is a common trend in oxide materials. It is worth mentioning that the density difference between *c*-MgSiO_3_ and *g*-MgSiO_3_ is much larger than that between *c*-Mg_2_SiO_4_ and *g*-Mg_2_SiO_4_.

**Table 1:** Density (g cm^–3^) Data for MgO–SiO_2_

	MgSiO_3_	Mg_2_SiO_4_
crystal	3.210^36^	3.220^37^
glass	2.740^7,9^	2.930^9^
liquid	2.511^38^	2.677^9^

#### Diffraction Data

**Figure 3:**
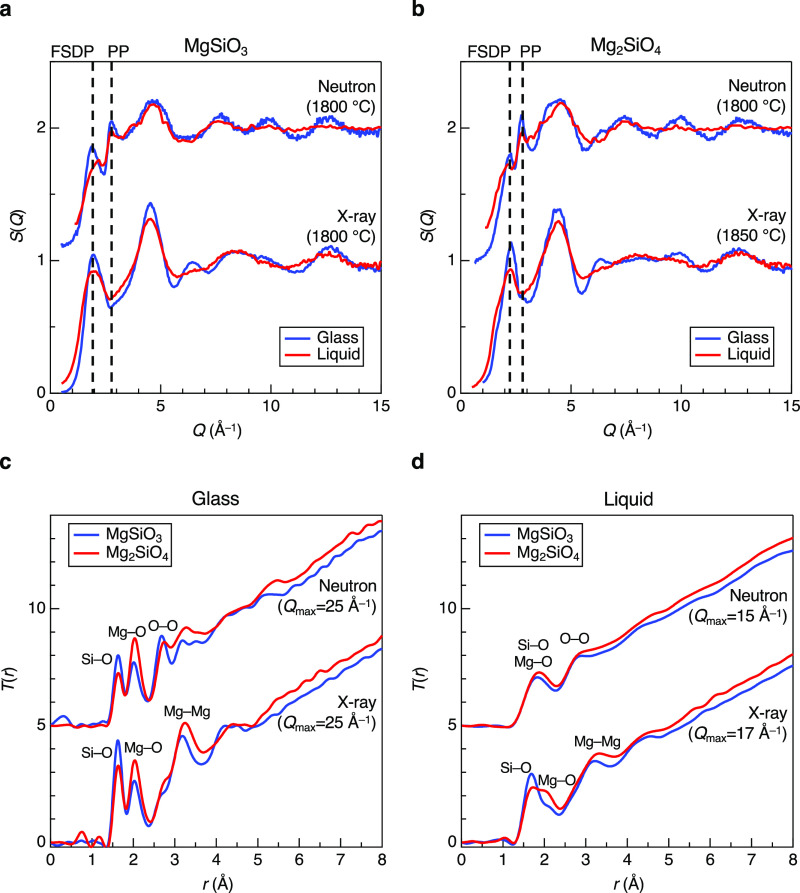
Diffraction data for MgO–SiO_2_ glasses and liquids. (a) Neutron (upper) and X-ray (lower) structure factors, *S*(*Q*) for *g*-^7^^,^^9^ and *l*-MgSiO_3_. (b) Neutron (upper) and X-ray (lower) structure factors, *S*(*Q*) for *g*-^7^^,^^9^ and *l*-Mg_2_SiO_4_. (c) Neutron (upper) and X-ray (lower) total correlation functions, *T*(*r*) for *g*-^7^^,^^9^ MgSiO_3_ (bule line) and Mg_2_SiO_4_ (red line). (d) Neutron (upper) and X-ray (lower) total correlation functions, *T*(*r*) for *l*-MgSiO_3_ (blue line) and Mg_2_SiO_4_ (red line). Dashed lines are guides for the eyes.

Figure 3 shows X-ray and neutron total structure factors, *S*(*Q*), for *g*-^9^ and *l*-MgSiO_3_ (a) and Mg_2_SiO_4_ (b), respectively. And also, Figure S2 shows X-ray total structure factors, *S*(*Q*), for *l*-*x*MgO–(100 – *x*)SiO_2_ were corrected using density data from Figure S1. The liquid data show broader features in comparison with the glass data due to the high temperature in Figure 3. A first sharp diffraction peak (FSDP)^39^ is observed at *Q* ∼ 2 and 2.2 Å^–1^ in the X-ray and neutron *S*(*Q*) for MgSiO_3_ and Mg_2_SiO_4_, respectively. An FSDP is considered the symbol of intermediate-range order which is composed of corner-sharing of SiO_4_ tetrahedra across the void. A principal peak (PP)^40^ is observed at *Q* ∼ 2.8 Å^–1^ in only the neutron *S*(*Q*) because the PP reflects the packing fraction of oxygen atoms,^41^ which neutrons are more sensitive to. The position of the FSDP in *g*-MgSiO_3_ is higher in *Q* than that of *g*-SiO_2_^9^ and that in *g*-Mg_2_SiO_4_ is higher in *Q* than that of *g*-MgSiO_3_ because the network comprised by the corner-sharing of SiO_4_ tetrahedra is broken down into MgSiO_3_ and Mg_2_SiO_4_ by the addition of MgO associated with the reduction of the cavity volume.^9^ On the other hand, the position of the PP in the neutron *S*(*Q*) is almost identical, but the peak heights for glasses are greater than those for liquids. This trend is consistent with density data because the PP reflects the packing fraction of the oxygen atoms as mentioned earlier.

The total correlation functions, *T*(*r*), for MgO–SiO_2_ glasses and liquids are shown in Figure 3. The real-space resolution in the glass data is better than that in the liquid data because we have measured the glass data with a wider *Q* range. In addition, the liquid structure is inherently more disordered than the glass structure, making peak assignment more difficult in the liquid data, as shown in Figure 3d. As can be seen in Figure 3c, we observe well-defined Si–O, Mg–O, and O–O peaks at approximately 1.63, 2.02, and 2.71 Å, respectively, but both the Mg–O and the O–O atomic distances in *g*-Mg_2_SiO_4_ are slightly longer than those in *g*-MgSiO_3_. We evaluated CNs using experimental and simulation data (Figure S3 and Tables S1 and S2), and it shows that the Si–O and Mg–O CNs are approximately 4 and 5 in both MgSiO_3_ and Mg_2_SiO_4_, although the Mg–O CNs in the glasses are slightly larger than those in the liquids, and those in Mg_2_SiO_4_ are larger than those in MgSiO_3_. These behaviors are in line with the behaviors of the PP in the neutron *S*(*Q*) and density data because the glasses are much denser than the liquids, and *g*- and *l*-Mg_2_SiO_4_ are denser than *g*- and *l*-MgSiO_3_. The average CN_Mg–O_ of DF–MD model of *g*-MgSiO_3_ shows 5.0 and the distribution of the value CN_Mg–O_ gives ^[4]^Mg (21.6%), ^[5]^Mg (55.9%), and ^[6]^Mg (22.5%) using the cutoff distance 2.60 Å. The previous results obtained by neutron diffraction, RMC, and empirical potential structure refinement (EPSR) show CN_Mg–O_ around 4.50,^42^ 4.50,^43^ and 4.48,^44^ respectively. Our DF–MD model has higher CN_Mg–O_ than those because a lot of ^[5]^Mg exist. On the other hand, ^[4]^Mg is predominant in other previous models; it might be our model slightly overestimates the Mg–O coordination.

#### Partial Structure and Short-Range Structure Derived from DF–MD Simulation

**Figure 4:**
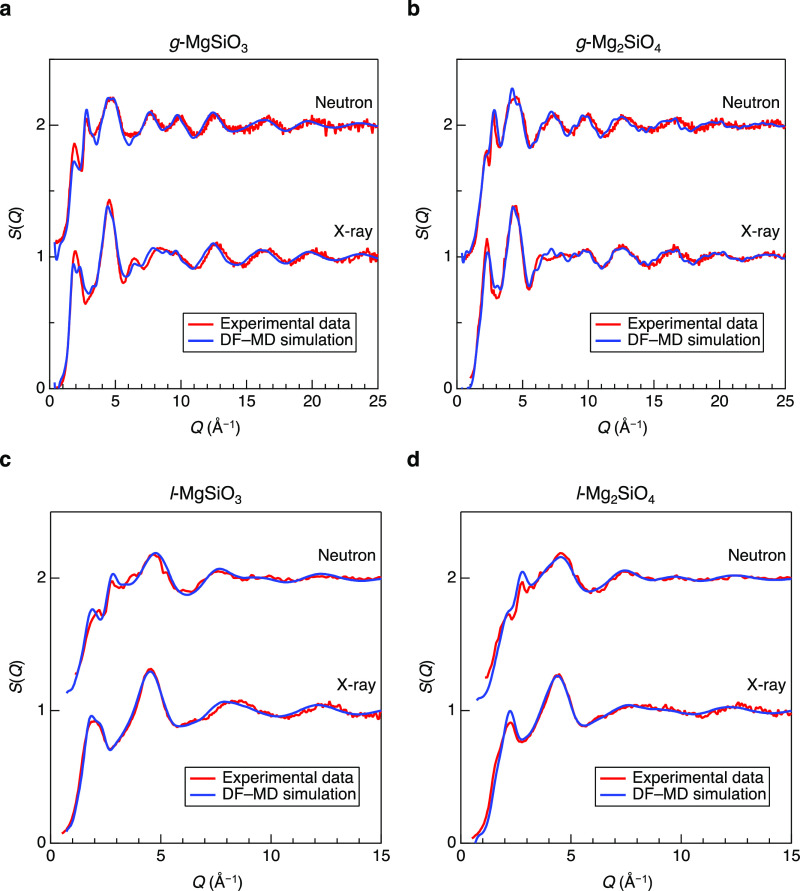
Neutron and X-ray total structure factors, *S*(*Q*), for *g*,*l*-MgO–SiO_2_ derived from DF–MD simulations (blue line) and experimental (red line) data.(a) Neutron (upper) and X-ray (lower) structure factors, *S*(*Q*) for *g*-^7^^,^^9^ MgSiO_3_. (b) Neutron (upper) and X-ray (lower) structure factors, *S*(*Q*) for *g*-^7^^,^^9^ Mg_2_SiO_4_. (c) Neutron (upper) and X-ray (lower) structure factors, *S*(*Q*) for *l*-MgSiO_3_. (d) Neutron (upper) and X-ray (lower) structure factors, *S*(*Q*) for *l*-Mg_2_SiO_4_.

**Figure 5:**
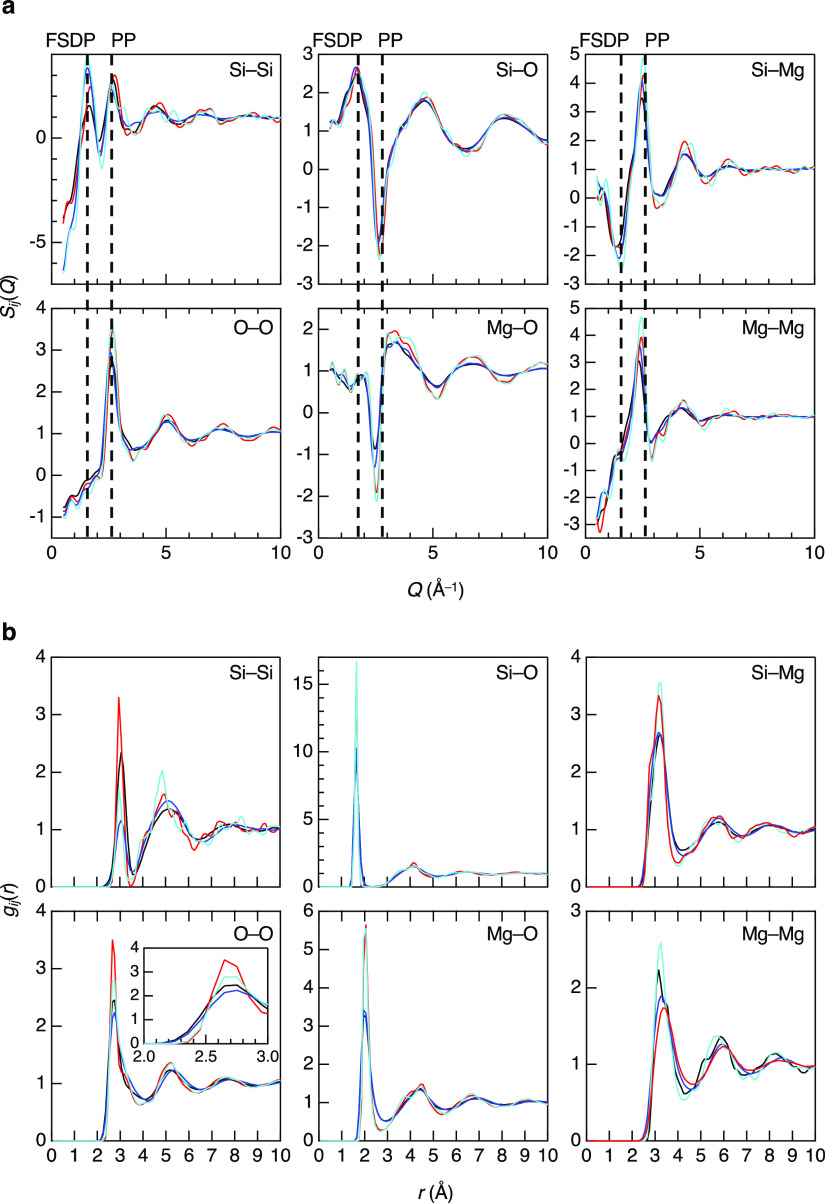
Partial structure for MgO–SiO_2_ glasses and liquids.(a) Partial structure factors, *S*_*ij*_(*Q*). (b) Partial pair distribution functions, *g*_*ij*_(*r*). Black, *l*-MgSiO_3_; red, *g*-MgSiO_3_; blue, *l*-Mg_2_SiO_4_; cyan, *g*-Mg_2_SiO_4_. Dashed lines are a guide to the eyes.

X-ray and neutron total structure factors, *S*(*Q*), for *g*-MgO–SiO_2_ and *l*-MgO–SiO_2_ derived from DF–MD simulations are shown in Figure 4. Figure 5a shows the partial structure factors, *S*_*ij*_(*Q*), for MgO–SiO_2_. The *S*_*ij*_(*Q*) except *S*_Si–Mg_(*Q*) exhibits a negative peak at the FSDP position. Similar behavior is found in 22.7R_2_O–77.3SiO_2_ glasses (R=Na and/or K).^45^ The cation–oxygen *S*_*ij*_(*Q*) (*S*_Si–O_(*Q*) and *S*_Mg–O_(*Q*)) exhibit a positive peak at the PP position, while the cation–cation *S*_*ij*_(*Q*) (*S*_Si–Si_(*Q*), *S*_Si–Mg_(*Q*), and *S*_Mg–Mg_(*Q*)) and *S*_O–O_(*Q*) exhibit a positive peak. The alkali–oxygen *S*_*ij*_(*Q*) in 22.7R_2_O–77.3SiO_2_ glasses do not exhibit such a negative peak at the PP position because alkali and magnesium have different valences, which results in different oxygen coordination numbers. Indeed, oxygen coordination numbers are mostly smaller than 5 in 22.7R_2_O–77.3SiO_2_ glasses. The partial pair distribution functions, *g*_*ij*_(*r*), for MgO–SiO_2_ derived from the DF–MD simulations are shown in Figure 5b. The first correlation peaks for the glasses are sharper than those of the liquids. The first correlation peaks of *g*_Si–Si_(*r*) in MgSiO_3_ are sharper than those in Mg_2_SiO_4_ and the first correlation peaks of *g*_Mg–Mg_(*r*) in Mg_2_SiO_4_ are sharper than those in MgSiO_3_, which reflect the composition difference between MgSiO_3_ (MgO–SiO_2_) and Mg_2_SiO_4_ (2MgO–SiO_2_).

It is confirmed that both the Si–O and Mg–O CNs derived from the DF–MD simulations are comparable to the experimental data. These behaviors suggest that there is no considerable structural difference in cation–oxygen coordination between MgSiO_3_ and Mg_2_SiO_4_ and between liquids and glasses. On the other hand, *g*_O–O_(*r*) shows significant differences between them, although the difference in oxygen atomic fractions between MgSiO_3_ (atomic fraction is 0.6) and Mg_2_SiO_4_ (atomic fraction is 0.57) is subtle. The O–O CNs for *g*-MgSiO_3_, *g*-Mg_2_SiO_4_, *l*-MgSiO_3_, and *l*-Mg_2_SiO_4_ are found to be 12.17, 12.70, 11.24, and 11.80, respectively. The difference between MgSiO_3_ and Mg_2_SiO_4_ and between liquids and glasses is large, which agrees well with the behavior of the PP in neutron *S*(*Q*). These results suggest that differences in packing fraction of oxygen atoms^46^ are an important parameter to understand the glass structure.

#### Three Body Correlations

**Figure 6:**
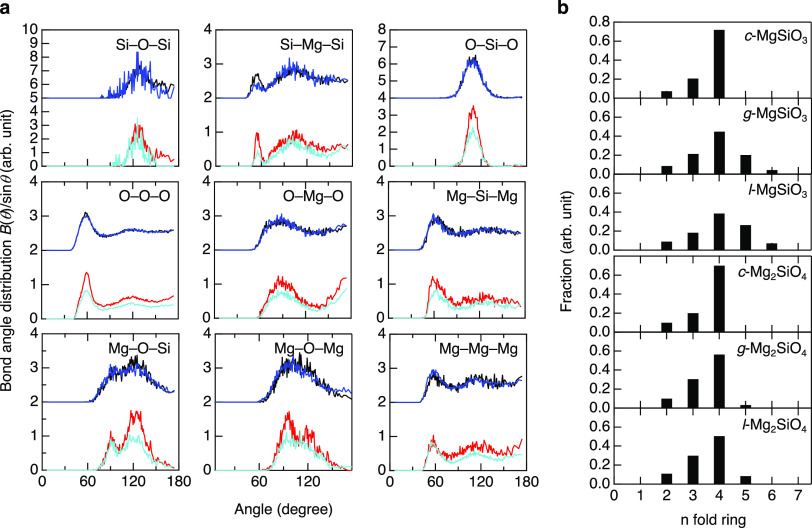
Analysis of intermediate-range structure in MgO–SiO_2_. (a) BADs. (b) King ring size distributions for −O–Si(Mg)–O– rings. Black, *l*-MgSiO_3_; red, *g*-MgSiO_3_; blue, *l*-Mg_2_SiO_4_; cyan, *g*-Mg_2_SiO_4_.

Figure 6a shows the bond angle distributions (BAD) for Mg–SiO_2_ glasses and liquids. It is worth mentioning that *l*-MgSiO_3_, *l*-Mg_2_SiO_4_, and *g*-Mg_2_SiO_4_ data are very similar, and only *g*-MgSiO_3_ exhibits a difference in fine structure in the Mg–O–Si and Mg–O–Mg BADs.

Especially, the Mg–O–Mg BAD exhibit that the MgO_*x*_ polyhedra of *g*-MgSiO_3_ have a unique connectivity because *c*-MgSiO_3_ shows only a single broad peak at ∼97° (no peak at ∼120°). The O–Mg–O BADs of *g*-MgSiO_3_ and *g*-Mg_2_SiO_4_ have two distinct peaks at ∼90 and ∼180°, which are very different from a single well-defined peak for O–Si–O (109°), suggesting that MgO_*x*_ polyhedra are octahedral, although the Mg–O CN is 5. The O–Mg–O BADs are rather similar to O–Er–O of *l*-Er_2_O_3_ (Er–O CN is 6.1)^47^ and O–Al–O of *g*-Al_2_O_3_ (Al–O CN is 4.7). Note that the Er_2_O_3_ is a nonglass-forming liquid and Al_2_O_3_ glass can be obtained only by electrochemical method^48^ since Al_2_O_3_ is classified into intermediate (nonglass former).^49^

#### Topological Analysis

From previous research,^9^ the addition of MgO decreases SiO_4_ tetrahedra rings because MgO worked as intermediate oxide. Especially, *g*-Mg_2_SiO_4_ has no SiO_4_ tetrahedra rings, and SiO_4_ monomer and Si_2_O_7_ dimer are predominant silicate species. In this research, we focused on the ring statistics for −O–Si(Mg)–O– rings in MgO–SiO_2_, and these data are shown in Figure 6b. Our ring statistics data are slightly different from those reported previously.^9^ We attribute this discrepancy to the different modeling approaches, i.e., RMC modeling in ref (9) vs a DF–MD simulation in our study. Fourfold rings are the dominant rings in all MgO–SiO_2_. Intriguingly, all ring size distributions are very similar in Mg_2_SiO_4_, while *g*- and *l*-MgSiO_3_ have larger-sized rings in comparison with *c*-MgSiO_3_. It is suggested that *g*- and *l*-MgSiO_3_ are topologically disordered,^50^ which is a typical behavior of high GFA glass, while *g*- and *l*-Mg_2_SiO_4_ are topologically very similar to *c*-Mg_2_SiO_4_. Table 2 summarizes the results of the polyhedral connections and *Q*^*n*^ analyses for MgO–SiO_2_. It is found that most of MgO–SiO_2_ are within the corner-sharing motif for SiO_4_–SiO_4_ connectivities, although small fractions of edge-sharing are observed in *g*-Mg_2_SiO_4_ and liquid MgO–SiO_2_. SiO_4_–MgO_*x*_ connectivities for *c*-MgSiO_3_ show a corner-sharing motif, too, but the fraction of edge-sharing is increased in *g*-MgSiO_3_ and shows the maximum value in *l*-MgSiO_3_ due to disorder. However, SiO_4_–MgO_*x*_ connectivities in Mg_2_SiO_4_ show completely different behavior. The fraction of edge-sharing is increased in *l*-Mg_2_SiO_4_ in comparison with *g*-Mg_2_SiO_4_, but the fraction of that in *c*-Mg_2_SiO_4_ shows the maximum value. Moreover, the ratio of corner-sharing and edge-sharing is exactly the same between SiO_4_–MgO_*x*_ connectivities and MgO_*x*_–MgO_*x*_ connectivities between *c*-Mg_2_SiO_4_ and *g*- and *l*-Mg_2_SiO_4_. The fraction of corner-sharing in MgO_*x*_–MgO_*x*_ connectivities in *c*-Mg_2_SiO_4_ is smaller than that in *g*-Mg_2_SiO_4_ and *l*-Mg_2_SiO_4_. On the other hand, MgO_*x*_–MgO_*x*_ connectivities in *c*-MgSiO_3_ show only edge-sharing, while the *g*-MgSiO_3_ shows a small fraction of edge-sharing in addition to corner-sharing and the fraction of edge-sharing slightly decreases in *l*-MgSiO_3_. Thus, the behavior is quite different between MgSiO_3_ and Mg_2_SiO_4_, and the latter shows similarity between *c*-Mg_2_SiO_4_ and *g*-/*l*-Mg_2_SiO_4_ because it is noted that the SiO_4_–SiO_4_ connectivities are subtle in *g*-/*l*-Mg_2_SiO_4_ owing to the breakdown of SiO_4_ network.

**Table 2:** Polyhedral Connections and *Q*^*n*^ Analyses for MgO–SiO_2_

	polyhedral connections				*Q^n^*				
		SiO_4_–SiO_4_	SiO_4_–MgO_*x*_	MgO_*x*_–MgO_*x*_	*Q*^0^	*Q*^1^	*Q*^2^	*Q*^3^	*Q*^4^
*c*-MgSiO_3_	corner	100	92.3	0	0	0	100	0	0
	edge	0	7.7	100					
	face	0	0	0					
*g*-MgSiO_3_	corner	100	82.7	65.9	4.9	22.6	44.1	21.4	7.0
	edge	0	17.3	30.7					
	face	0	0	3.4					
*l*-MgSiO_3_	corner	98.5	77.1	69.6	5.8	20.3	44.3	25.7	3.9
	edge	1.5	22.5	27.9					
	face	0	0.4	2.5					
*c*-Mg_2_SiO_4_	corner	0	66.7	66.7	100	0	0	0	0
	edge	0	33.3	33.3					
	face	0	0	0					
*g*-Mg_2_SiO_4_	corner	96.6	79.3	71.5	39.6	43.8	16.6	0	0
	edge	3.4	20.7	27.4					
	face	0	0	1.1					
*l*-Mg_2_SiO_4_	corner	98.4	76.2	68.5	37.1	45.2	16.2	1.4	0.1
	edge	1.6	23.2	28.3					
	face	0	0.6	3.2					

*Q*^*n*^ distributions summarized in Table 2 provide us with connectivities of SiO_4_ polyhedra. *c*-MgSiO_3_ shows quite unique connectivity, because we can observe only *Q*^2^ chain network. Indeed, it is demonstrated that SiO_4_ tetrahedra form a corner-sharing chain network and MgO_*x*_ polyhedra form only an edge-sharing network, which form a layer structure in *c*-MgSiO_3_. More than 50% of the *Q*^2^ chain transforms into *Q*^1^ and *Q*^3^ in both *g*-MgSiO_3_ and *l*-MgSiO_3_, suggesting that the structural transformation between *c*-MgSiO_3_ and *g*-/*l*-MgSiO_3_ requires quite significant structural modifications. On the other hand, *c*-Mg_2_SiO_4_ shows only *Q*^0^ because the SiO_4_ tetrahedra are isolated. Moreover, the fractions of *Q*^0^ in *g*- and *l*-Mg_2_SiO_4_ are decreased to less than 40% and *Q*^1^ (Si_2_O_7_^6–^ dimers^7^) is dominant (43.8% in glass and 45.2% in liquid), while the fractions of *Q*^2^ are about 16%. In addition, a small fraction of *Q*^3^ (1.4%) and *Q*^4^ (0.1%) is observed in *l*-Mg_2_SiO_4_. It is suggested from these behaviors that the transformation from *g*/*l*-Mg_2_SiO_4_ into *c*-Mg_2_SiO_4_ seems to be easier than that in MgSiO_3_ because only the breakdown of chains or dimers is required while the formation of chains is required in the transformation from *g*/*l*- MgSiO_3_ into *c*-MgSiO_3_. The average *Q*^*n*^ values of MgO–SiO_2_ are 2.00 (*c*-MgSiO_3_), 2.03 (*g*-MgSiO_3_), 2.02 (*l*-MgSiO_3_), 0 (*c*-Mg_2_SiO_4_), 0.77 (*g*-Mg_2_SiO_4_), and 0.82 (*l*-Mg_2_SiO_4_). Both *g-* and *l*-SiO_2_ with high GFA have the value of that average *Q*^*n*^ are almost 4.0, which suggested that the number of average *Q*^*n*^ is an indicator of GFA.

**Figure 7:**
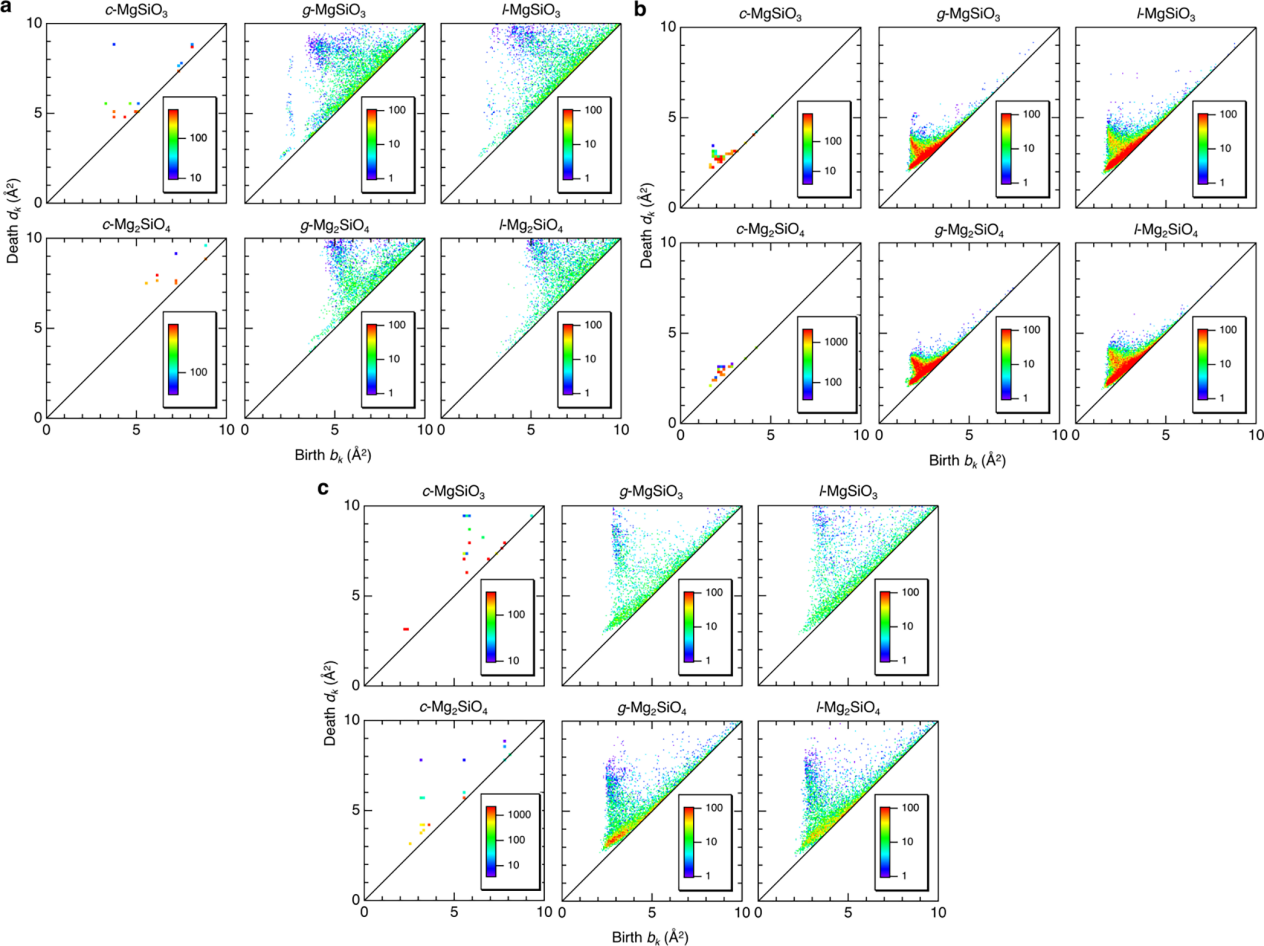
Topological analysis for MgO–SiO_2_. (a) Si-centric PD_1_, (b) O-centric PD_1_, and (c) Mg-centric PD_1_.

Figure 7a shows the Si-centric persistence diagrams, PD_1_s. It is well-known that *g*-SiO_2_ shows a prominent vertical profile along with the death axis at *b*_*k*_ ∼ 2.2 Å^2^ in both the Si-centric and O-centric PD_1_s due to the formation of SiO_4_ tetrahedral network with corner-sharing of oxygen atoms.^51,52^ Similar profiles are only observed in the Si-centric PD_1_ for *g*- and *l*-MgSiO_3_ at *b*_*k*_ ∼ 2.4 Å^2^, but *c*-MgSiO_3_ does not show such a profile since *c*-MgSiO_3_ has only a *Q*^2^ chain network, which is not three-dimensional. Mg_2_SiO_4_ does not show such a profile, either, because there is almost no *Q*^3^ or *Q*^4^ three-dimensional SiO_4_ network. The O-centric PD_1_s are shown in Figure 7b. The small death profiles are observed along with the diagonal in PD_1_s because the death values reflect significantly high packing of oxygen atoms and high density. It is found that the death value is a maximum in *l*-MgSiO_3_ (minimum density) and a minimum in *c*-MgSiO_3_ (maximum density). Figure 7c shows Mg-centric PD_1_s. The PD_1_s for *g*-MgSiO_3_ and *g*-Mg_2_SiO_4_ have a profile along with the death axis at *b*_*k*_ ∼ 3.0 Å^2^, which are the signature for the formation of the Mg–O network. The PD_1_s for *c*-Mg_2_SiO_4_ have a profile at the same *b*_*k*_ position, while *c*-MgSiO_3_ does not have such a profile because of the absence of well-defined three-dimensional Mg–O network. Moreover, it is suggested that all of the liquid data are very similar to glass data and that *c-*Mg_2_SiO_4_ data are very similar to *g*-Mg_2_SiO_4_, but *c*-MgSiO_3_ data are very different from *g*/*l*-MgSiO_3_ data. This trend is consistent with ring size distributions, demonstrating that we can see similarity in homology in Mg_2_SiO_4_, but the homology of *c*-MgSiO_3_ is quite different from that of *g*- and *l*-MgSiO_3_.

#### Electronic Structures

**Figure 8:**
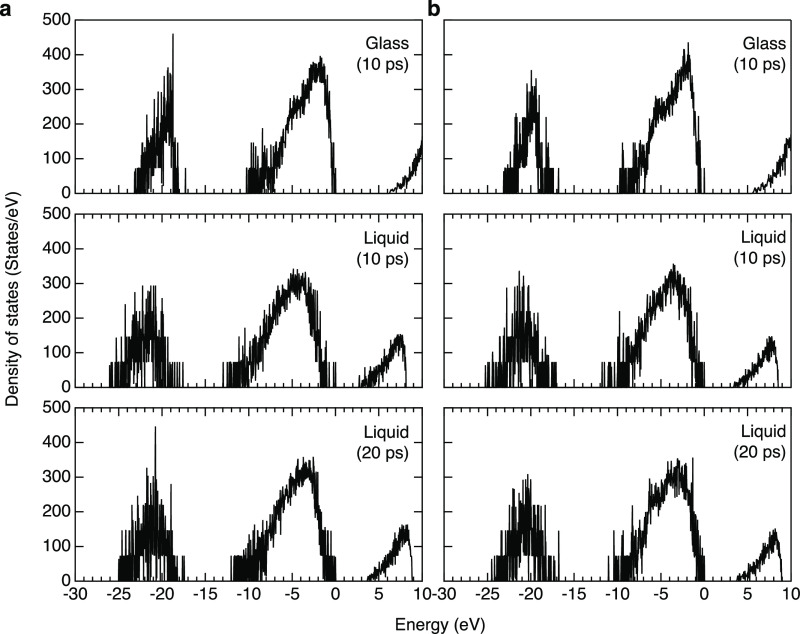
Electronic structure of MgO–SiO_2_ glasses and liquids. Electron DOSs for (a) MgSiO_3_ and (b) Mg_2_SiO_4_ glasses and liquids calculated by DF–MD simulations employing PBE0 (0.3).

**Figure 9:**
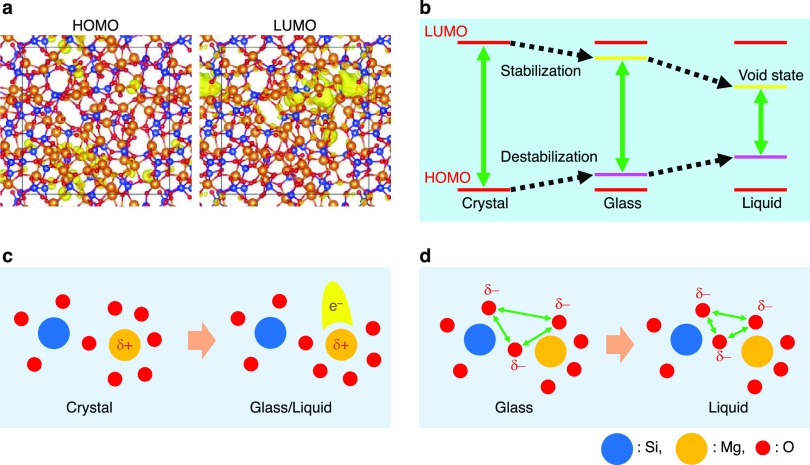
Behaviors of HOMO and LUMO in MgO–SiO_2_.(a) Isosurface plots of the partial charge density around the HOMO and the LUMO levels for *g*-MgSiO_3_. (b) Schematic illustration for HOMOs and LUMOs in crystals, glasses, and liquids. (c) Schematic illustration of LUMO in glasses and liquids. (d) Schematic illustration for electron repulsions in liquids.

Figure 8 shows electron density of states (DOS) for *g*-and *l*-MgO–SiO_2_ calculated employing PBE0^53^ with a fraction of exact exchange of α = 0.3, which will be referred to as PBE0 (0.3) below. It is known that GGA–PBE underestimates the band gap, and we performed several benchmark tests for crystalline MgO, SiO_2_ (α-quartz), MgSiO_3_, and Mg_2_SiO_4_ (see Table S3) and confirmed that PBE0 (0.3) shows the best agreement with experimental data; hence, we compare GGA–PBE (blue) and PBE0 (0.3) (red) in Figure S4. It is suggested from the DF–MD calculations that the lowest unoccupied molecular orbitals (LUMOs) are 3*s* orbitals and free electron-like state at the void sites near magnesium atoms (see Figure 9a for *g*-MgSiO_3_ as a typical example and Figure S5 for *l*-MgSiO_3_ and *g*-/*l*-Mg_2_SiO_4_) arising from a decreased oxygen coordination, and the highest occupied molecular orbitals (HOMOs) are oxygen’s 2*p* orbital states. These behaviors are in line with our previous study on CaO–Al_2_O_3_ glass^54^ but very different from α-quartz, *c*-MgO, *c*-MgSiO_3_, and *c*-Mg_2_SiO_4_, in which LUMOs and HOMOs are oxygen’s 3*s* and 2*p* orbitals, respectively. Electron band gaps calculated by PBE0 (0.3) are found to be 7.97, 6.30, and 2.71 (10 ps)/3.69 (20 ps) eV, for *c*-, *g*, and *l*-MgSiO_3_ and 8.37, 5.64, and 3.43 (10 ps)/3.81 (20 ps) eV, for *c*-, *g*, and *l*-Mg_2_SiO_4_. Note that liquid data have more fluctuations due to the high temperature. It is found that band gap values become small in the order of crystal, glass, and liquid (see Figure 9b) and the band gap of *g*-Mg_2_SiO_4_ is narrower than that of *g*-MgSiO_3_. We discuss these behaviors in Figure 9. The LUMO levels of glasses can be stabilized due to void site arising from a decreased oxygen coordination from six in the crystals to five in the glasses (Figure 9c). The LUMO levels of the liquid can be more stabilized due to the high temperature. HOMO can be destabilized in glasses due to inherent structural disorder, especially between oxygen atoms. This feature is enhanced in the liquid due to high temperature (see Figure 9d) manifested by decreased minimum oxygen–oxygen atomic distances shown in the inset of Figure 5b.

## Conclusions

In this article, we have discussed the atomic and electronic structures of MgSiO_3_ and Mg_2_SiO_4_ to understand the network topology and relationship between structure and GFA. The density measurement at the ISS confirmed that our previous estimated density for *l*-Mg_2_SiO_4_ is very close to experimental data. The packing of oxygen atoms in Mg_2_SiO_4_ is larger than that in MgSiO_3_, and that of the glasses is larger than that of the liquids. Diffraction measurements and DF–MD simulations demonstrated that the packing of oxygen atoms is an important structural descriptor to understand the difference between MgSiO_3_ and Mg_2_SiO_4_ and between glass and liquid. The analysis of electronic and topological structures reasonably explained the behaviors of electron band gaps and topological similarity in crystals, glasses, and liquids. These results suggest that an electronic state does not change quite a lot between MgSiO_3_ and Mg_2_SiO_4_, also the topological similarity between crystalline (*c*)- and *g*-(*l*-) Mg_2_SiO_4_ is the signature of low GFA and high GFA *g*-(*l-*) MgSiO_3_ shows a unique glass topology, which is different from *c-*MgSiO_3_. This means the atomic structure in terms of network topology is an important factor to understand GFA. We demonstrated that systematic comparison among crystal, glass, and liquid is important to understand the nature and glass and liquid. The utilization of containerless techniques and understanding of behavior of oxygen atoms, as well as network topology, provide us with crucial information to discuss glass-forming ability.

## Data Availability

The data sets used and/or analyzed during the current study available from the corresponding author on reasonable request.

## Supporting Information Available

Experimental and computational details; density data of *l*– *x*MgO–(100 – *x*)SiO_2_; X-ray total structure factors, *S*(*Q*), for *l*– *x*MgO–(100 – *x*)SiO_2_; pair function analysis; electron density of states; Isosurface plots of the partial charge density; coordination numbers obtained by pair function analysis; coordination number obtained by DF–MD; electron band gaps of GGA–PBE and PBE0 (PDF).
