# Supplementary material for: Atomic and Electronic Structure in MgO–SiO2
Source: J Phys Chem A. 2024 Jan 18;128(4):716–26. doi: 10.1021/acs.jpca.3c05561 (PMC10839830; doi:10.1021/acs.jpca.3c05561)
Supplement: Supplementary file 1 — jp3c05561_si_001.pdf [file jp3c05561_si_001.pdf]

**Supporting Information**

**Atomic and Electronic Structure in $MgO–SiO_2$**

*Yuta Shuseki*[a,b], *Shinji Kohara*[b*], *Tomoaki Kaneko*[c], *Keitaro Sodeyama*[b], *Yohei Onodera*[b], *Chihiro Koyama*[d], *Atsunobu Masuno*[a,b], *Shunta Sasaki*[e], *Shohei Hatano*[e], *Motoki Shiga*[f,g,h], *Ippei Obayashi*[i], *Yasuaki Hiraoka*[j], *Junpei T. Okada*[k], *Akitoshi Mizuno*[l], *Yuki Watanabe*[m], *Yui Nakata*[m], *Koji Ohara*[n], *Motohiko Murakami*[o], *Matthew G. Tucker*[p], *Marshall T. McDonnell*[q], *Hirohisa Oda*[d] *and Takehiko Ishikawa*[r]

[a] *Graduate School of Engineering, Kyoto University, Kyoto 615-8520, Japan*

[b] *Center for Basic Research on Materials, National Institute for Materials Science (NIMS), Tsukuba, Ibaraki 305-0047, Japan*

[c] *Department of Computational Science and Technology, Research Organization for Information Science and Technology (RIST), Tokyo 105-0013, Japan*

[d] *Human Spaceflight Technology Directorate, Japan Aerospace Exploration Agency (JAXA), Tsukuba, Ibaraki 305-8505, Japan*

[e] *Graduate School of Science and Technology, Hirosaki University, Hirosaki, Aomori 036-8561, Japan*

[f] *Unprecedented-Scale Data Analytics Center, Tohoku University, Sendai, Miyagi 980-8578, Japan*

[g] *Graduate School of Information Science, Tohoku University, Sendai, Miyagi 980-8579, Japan*

[h] *RIKEN Center for Advanced Intelligence Project, Tokyo 103-0027, Japan*

[i] *Center for Artificial Intelligence and Mathematical Data Science, Okayama University, Okayama 700-8530, Japan*

[j] *Institute for the Advanced Study of Human Biology (WPI-ASHBi), Kyoto University, Kyoto* 606-8303*, Japan*

[k] *Institute for Materials Research, Tohoku University, Sendai, Miyagi 980-8577, Japan*

[l] *National Institute of Technology, Hakodate College, Hakodate, Hokkaido 042-8501, Japan*

[m] *Advanced Engineering Services Co., Ltd., Tsukuba, Ibaraki* 305-0032*, Japan*

[n] *Faculty of Materials for Energy, Shimane University, Matsue, Shimane 690-8504, Japan*

[o] *Department of Earth Sciences, ETH Zürich, Zürich 8092, Switzerland*

[p] *Neutron Scattering Division, Oak Ridge National Laboratory, Oak Ridge, Tennessee 37831, United States*

[q] *Computer Science and Mathematics Division, Oak Ridge National Laboratory, Oak Ridge, Tennessee 37830, United States*

[r] *Institute of Space and Astronautical Science, Japan Aerospace Exploration Agency (JAXA), Tsukuba, Ibaraki 305-8505, Japan*

[*]Corresponding author: Shinji Kohara, email:KOHARA.Shinji@nims.go.jp

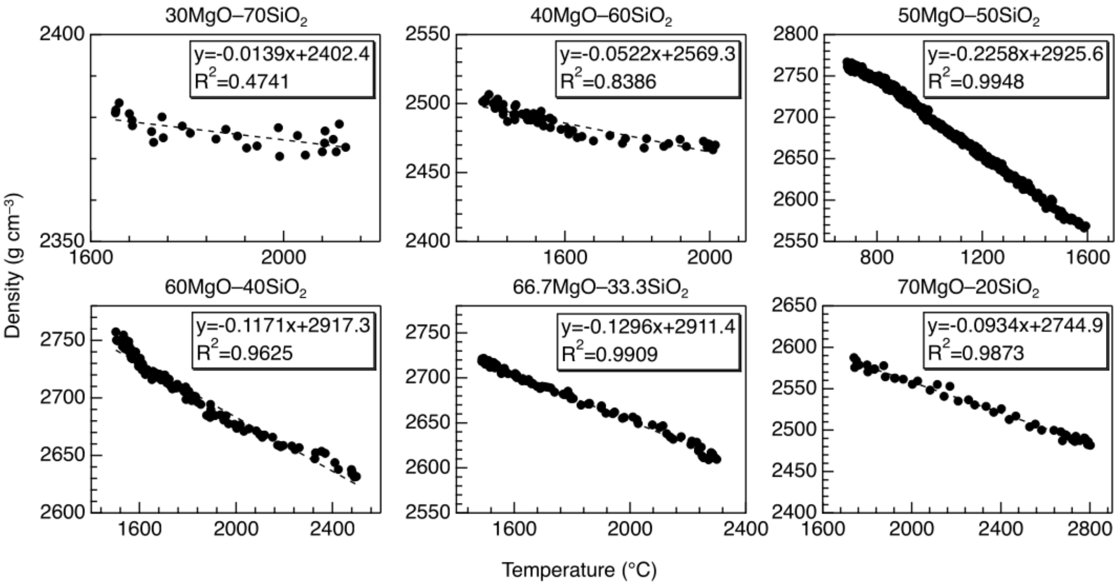


**Fig. S1** Measured density of *l*-*x*MgO–(100-*x*)$SiO_2$ as a function of temperature.

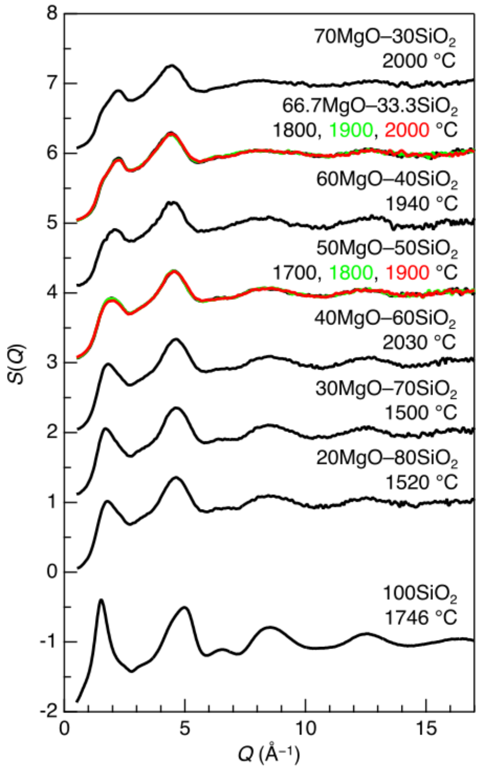


**Fig. S2** X-ray total structure factors, $S(Q)$, for $l$-$x$MgO–(100-$x$)$SiO_2$ and $l$-$SiO_2$[1].

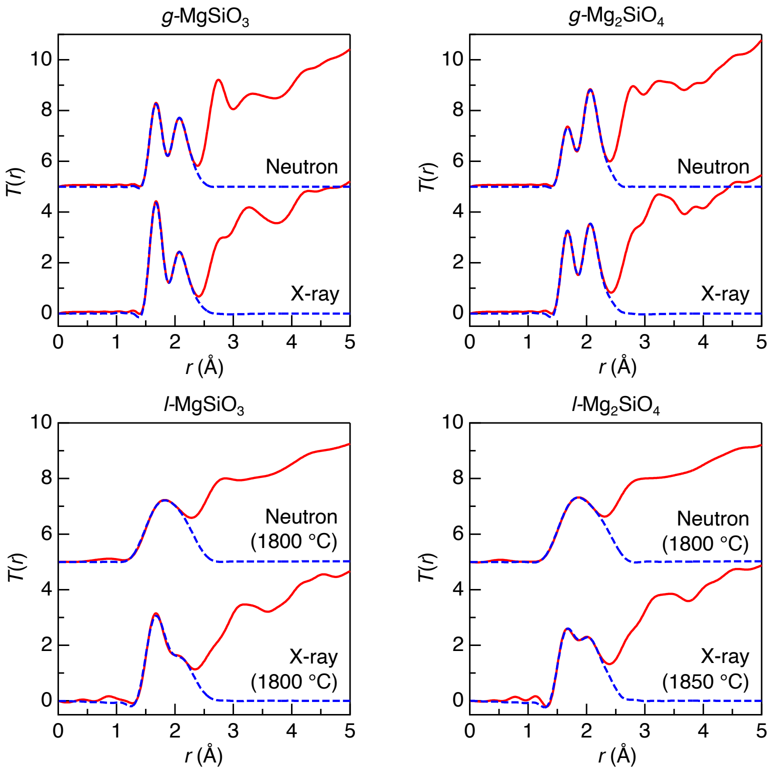


**Fig. S3** Pair function analysis of total correlation functions $T(r)$. Red; experimental data, bule; pair function.

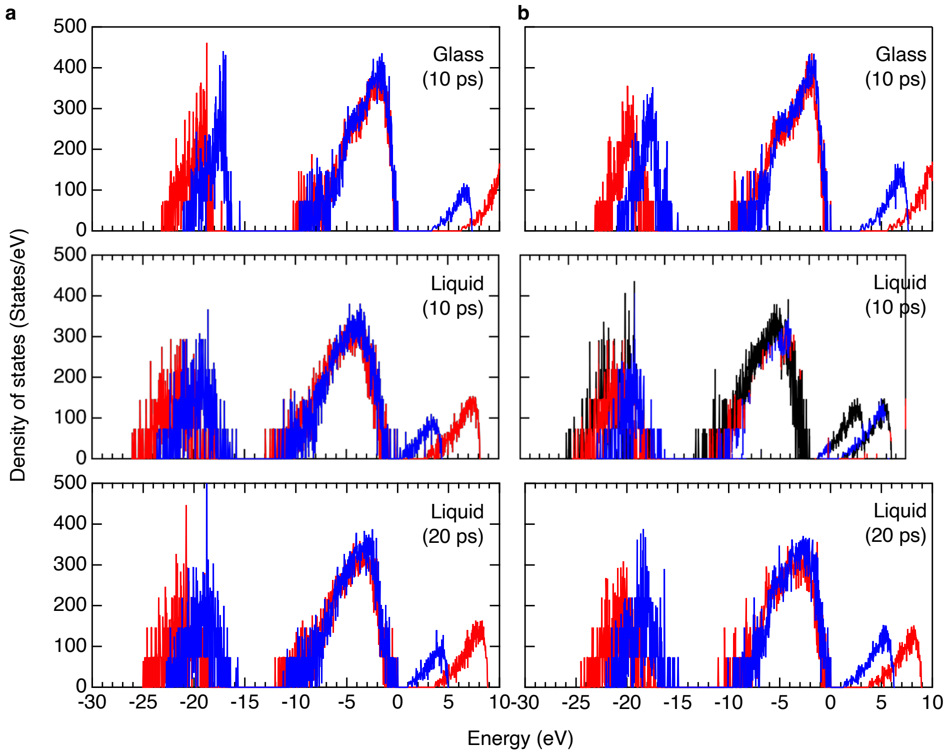


**Fig. S4** Electron density of states for **a** *g*- and *l*-$MgSiO_3$ and **b** *g*- and *l*-$Mg_2SiO_4$ calculated by DF–MD simulations. Red; PBE0 (0.3), blue; GGA–PBE.

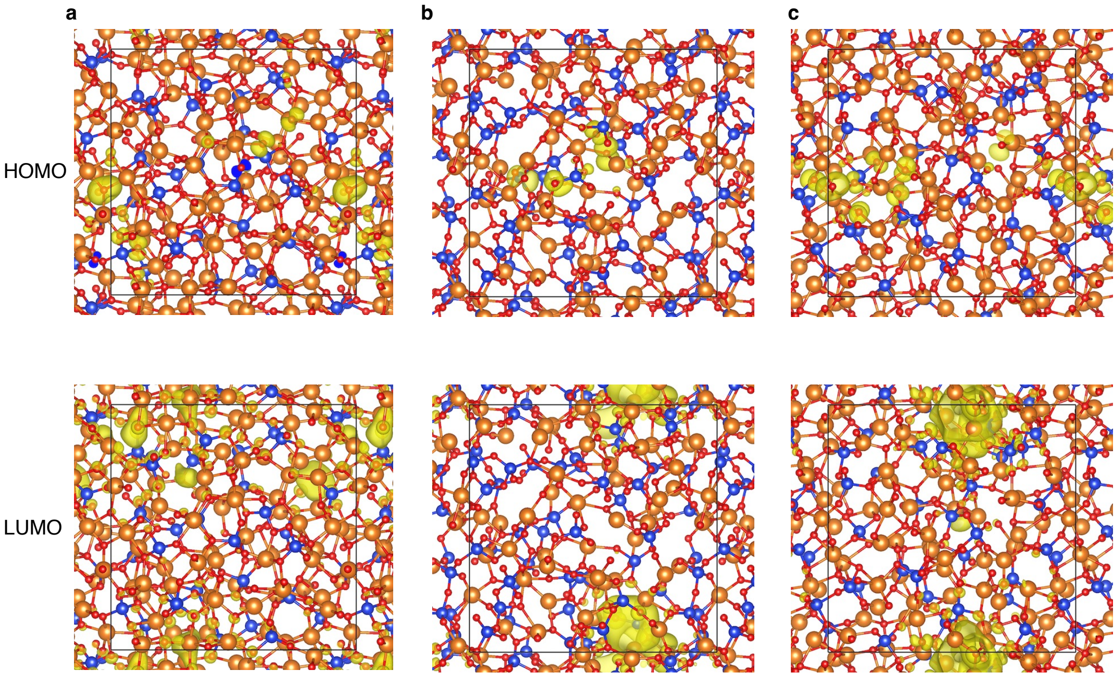


**Fig. S5** Isosurface plots of the partial charge density around the HOMO and the LUMO levels for **a** *g*-$Mg_2SiO_4$ density of states, **b** *l*-$MgSiO_3$ (10 ps) and **c** *l*-$Mg_2SiO_4$ (10 ps).

**Table S1** Coordination numbers obtained by pair function analysis of X-ray and neutron total correlation functions *T*(*r*).

| | | $N_{Si–O}$ | $N_{Mg–O}$ |
|---|---|---|---|
| *l*-$MgSiO_3$ | neutron | 3.85 | 4.8<br>(3.7+1.1) |
| | X-ray | 3.85 | 4.8<br>(4.0+0.8) |
| *g*-$MgSiO_3$ | neutron | 3.95 | 5.0<br>(4.5+0.5) |
| | X-ray | 3.95 | 5.0<br>(4.5+0.5) |
| *l*-$Mg_2SiO_4$ | neutron | 3.85 | 4.8<br>(3.55+1.25) |
| | X-ray | 3.85 | 4.8<br>(3.9+0.9) |
| *g*-$Mg_2SiO_4$ | neutron | 4.00 | 5.0<br>(4.4+0.6) |
| | X-ray | 3.95 | 5.0<br>(4.5+0.5) |

**Table S2** Coordination numbers obtained by DF–MD models.

| DF–MD model | $N_{Si–O}$ (cut off 1.9 Å) | $N_{O–O}$ (cut off 4.0 Å) | $N_{Mg–O}$ (cut off 2.8 Å) |
|---|---|---|---|
| *l*-$MgSiO_3$ | 3.84 | 11.24 | 4.82 |
| *l*-$Mg_2SiO_4$ | 3.88 | 11.82 | 4.93 |
| *g*-$MgSiO_3$ | 4.00 | 12.17 | 5.19 |
| *g*-$Mg_2SiO_4$ | 4.03 | 12.70 | 5.15 |

**Table S3** Electron band gaps (eV) for several crystals calculated by GGA–PBE and PBE0.

| Method | MgO | α-quartz | $MgSiO_3$ | $Mg_2SiO_4$ |
|---|---|---|---|---|
| Exp. | 7.7[2]/7.8[3] | 9.34-9.55[4] | 8.9[5] | 8.4[6]/8.8[5] |
| GGA–PBE | 4.82779 | 5.53158 | 4.88708 | 5.27906 |
| PBE0 (0.25) | 7.33461 | 8.06809 | 7.44759 | 7.85018 |
| PBE0 (0.30) | 7.84888 | 8.58155 | 7.96749 | 8.37234 |
| PBE0 (0.35) | 8.36677 | 9.09690 | 8.48946 | 8.89693 |

Values in brackets show fractions of the exact exchange term for the exchange correlation functional.

## References


1. Q. Mei *et al*. Structure of liquid $SiO_2$: a measurement by high-energy X-ray diffraction. *Phys. Rev. Lett.* **98**, 057802 (2007).
2. S. Adachi, *Optical Properties of Crystalline and Amorphous Semiconductors: Numerical Data and Graphical Information* (Kluwer Academic, Dordrecht, 1999).
3. Roessler, D. M. & Walker, W. C. Electronic spectrum and ultraviolet optical properties of crystalline MgO. *Phys. Rev.* **159**, 733–738 (1967).
4. Tan, G. L. *et al.* Optical properties and London dispersion interaction of amorphous and crystalline $SiO_2$ determined by vacuum ultraviolet spectroscopy and spectroscopic ellipsometry. *Phys. Rev. B* **72**, 205117 (2005).
5. Nitsan, U. & Shankland, T. J. Optical properties and electronic structure of mantle silicates. *Geophys. J. Int.* **45**, 59–87 (1976).
6. Shankland, T. J. Band gap of forsterite. *Science* **161**, 51–53 (1968).
